# Anti-angiogenic therapies in brain metastases

**DOI:** 10.1007/s12254-018-0384-2

**Published:** 2018-02-02

**Authors:** Anna S. Berghoff, Matthias Preusser

**Affiliations:** Department of Medicine I and Comprehensive Cancer Center Central Nervous System Tumours Unit (CCC-CNS), Medical University of Vienna, Währinger Gürtel 18–20, 1090 Vienna, Austria

**Keywords:** CNS metastases, Systemic treatment, Bevacizumab, Asymptomatic, Symptomatic

## Abstract

Brain metastases are a major challenge in modern oncology, as treatment options upon the diagnosis of symptomatic brain metastases are limited. Neo-angiogenesis was identified as a hallmark of brain metastasis development and inhibition using anti-angiogenic therapy might therefore be an experimental promising preventive as well as therapeutic approach. The current review will summarize the current available data on the efficacy of neo-angiogenic therapies in patients with brain metastases.

## Take home message

Neo-angiogenesis is a hallmark of the brain metastasis cascade. Anti-angiogenic therapy has shown promising effects as a preventive as well as a therapeutic approach in the treatment of patients with brain metastases.

## Introduction

Brain metastases are a major clinical challenge, as up to 40% of patients with metastatic cancer develop symptomatic brain metastases during their clinical course of disease [1, 2]. Lung cancer, followed by breast cancer and melanoma, are the most frequent underlying histologies. However, other histologies like renal carcinoma and colorectal cancer were shown to present, with rising incidences of symptomatic brain metastases in the last decade [3]. Local therapies are currently still the main treatment backbone in patients with symptomatic brain metastases; however, the value of systemic targeted or immune-modulating therapies has increased, especially in patients with oligo- to asymptomatic disease [4]. Further, prevention of symptomatic brain metastases by targeted treatment has become a major field of interest, as brain metastases are frequently a life-limiting complication with an enormous impact on the quality of life [5].

Anti-angiogenic therapies including monoclonal antibodies targeting the vascular endothelia growth factor (VEGF) like bevacizumab, antibodies targeting the VEGF receptors like ramucirumab, and VEFR receptor tyrosine kinase inhibitors like sunitinib, sorafenib, or nintedanib have been widely studied in several entities frequently causing brain metastases, like lung cancer or triple-negative breast cancer, and represent an important treatment backbone. Therefore, the following review will focus on the biology of neo-angiogenesis in brain metastasis development and the subsequent treatment options with anti-angiogenic therapies in this particular clinical context.

## Neo-angiogenesis is a specific hallmark of brain metastasis development

Preclinical data of a brain metastasis mouse model using repetitive multi-photon in vivo imaging revealed that neo-angiogenesis is a specific hallmark of non-small cell lung (NSCLC) cancer brain metastases and pivotal for macrometastasis outgrowth [6]. In detail, induction of neo-angiogenesis was essential in the step from single perivascular cell to mirco- and macrometastasis, as anti-angiogenic treatment with the VEFG inhibitor bevacizumab inhibited this process effectively (as compared with placebo) and resulted in the prevention of macrometastasis outgrowth. In contrast, melanoma brain metastases presented with a rather co-optive growth pattern along pre-existing vascular structures and less neo-angiogenesis [6]. In line with this, macrometastasis development was not influenced by the anti-angiogenic treatment with VEFG inhibitor bevacizumab, suggesting that the growth pattern of brain metastases differs according to their histology. Indeed, different growth patterns including differences in the neo-angiogenesis were observed in human autopsy specimens, supporting the idea that neo-angiogenesis and well-demarked growth are a specific hallmark of NSCLC brain metastases, while melanoma brain metastases present more frequently with a co-optive growth pattern and less neo-angiogenesis [7, 8].

The importance of neo-angiogenesis in brain metastasis development is further supported by the preventive potential of anti-angiogenic therapies in patients with a high risk of brain metastasis development. Post hoc analysis of the AVAIL data (Platin-based chemotherapy plus/minus bevacizumab therapy as first-line therapy in newly diagnosed metastatic/advanced NSCLC) showed that patients treated with bevacizumab had a statistically significantly lower chance of brain metastases as the first site of recurrence [9]. Clinical data for the preventive potential of neo-angiogenic therapies in other histologies like breast cancer or renal cell cancer are conflicting and could be potentially explained by a higher variability of neo-angiogenesis observed in brain metastases of these histologies [8]. Post hoc analysis of the AVADO and AVEREL (Chemotherapy plus/minus bevacizumab therapy as first-line therapy in newly diagnosed metastatic/advanced breast cancer) showed no statistically significant reduction of brain metastasis occurrence as the first site of relapse [9]. Preclinical data suggest that indeed only combined inhibition of VEGF and angiopoetin-2 might result in a reduction of brain metastasis occurrence in breast cancer and might therefore be interesting for further clinical investigation [10]. A single-center retrospective study analyzed the preventive potential of anti-angiogenic therapies (sorafenib, sunitinib, bevacizumab) in patients with metastatic renal cell carcinoma [11]. No preventive potential of the anti-angiogenic therapies was shown, further supporting the notion that extensive neo-angiogenesis is a specific hallmark of NSCLC brain metastasis.

## Efficacy of anti-angiogenic therapies in established, asymptomatic brain metastases

The risk of CNS bleeding was a major concern upon the introduction of anti-angiogenic therapies and resulted in the frequent exclusion of brain metastasis patients from clinical trials [5, 12]. However, several subsequent studies proved that there is no increased safety issue, as frequency of bleeding as well as other side effects were not accumulated in brain metastasis patients [12–14]. In line with this, the rate of bevacizumab-related adverse events was similar among patients with (21%) and without (20%) brain metastases [15]. Nevertheless, due to the systematic exclusion of brain metastases patients in the majority of phase III trials, only limited knowledge on the efficacy of anti-antigenic therapies in brain metastasis patients is available [5].

Some phase II trials as well as retrospective analyses investigated the efficacy of bevacizumab-based treatments in patients with asymptomatic NSCLC brain metastases (Table 1) [15, 16]. Here, intracranial response rates of bevacizumab in combination with carboplatin and paclitaxel (61.2%) or in combination with erlotinib (12.5%; unselected cohort) similar to the expected extracranial response rate were observed [16]. Median progression-free survival ranged from 6.3 to 6.7 months and overall survival from 12 (erlotinib combination; unselected for EGFR status) to 16 (carboplatin and paclitaxel combination) months, and was therefore shown to be similar between patients without brain metastases and patients with asymptomatic brain metastases [15–17]. For most other entities, only case reports or small case series have focused on the efficacy of anti-angiogenic therapies. Progression-free survival after treatment with paclitaxel and bevacizumab ranged from 6 to 11 months in a case series of five patients with breast cancer brain metastases [18]. A case series of 16 patients suffering from newly diagnosed, untreated brain metastases from renal cell carcinoma treated with first-line sunitinib presented with a median time to progression of 2.3 months, a median overall survival of 6.3 months, and an overall good tolerability of sunitinib [19]. Similarly, analysis of the expanded access program of sunitinib in renal cell carcinoma brain metastasis patients (*n* = 231) revealed a median progression-free survival of 5.6 months and a median overall survival of 9.2 months [20]. Further, some case reports also suggest a clinical efficacy in the rare event of colorectal carcinoma brain metastases. Here, a median overall survival of 20.6 months (range 7–42 months) after bevacizumab-based treatment was observed in patients with colorectal brain metastases (*n* = 5) [21].

## Anti-angiogenic therapies as palliative treatment for patients with symptomatic brain metastases

Local therapies including stereotactic radiosurgery, whole-brain radiotherapy, and neurosurgical resection are important treatment backbones in patients with symptomatic brain metastases [4]. The local therapy approach is chosen depending on the number and size of brain metastases, the performance status of the patient, and the extent of the extracranial disease [4]. Intracranial recurrence, either locally or distant, after first-line local therapy is frequently observed and the life-limiting progression in the majority of patients [3]. Upon recurrence, neurosurgical resection can be considered as a salvage therapy if immediate symptom relief due to mass effect of the surrounding peritumoral edema is needed. Further, re-radiation can also be considered carefully in previously radiated lesions [22]. However, radiation necrosis is a potential complication and associated with significant neurological morbidity due to the mass effect of the large peritumoral edema. High steroid doses are frequently needed to control symptoms, but cause iatrogenic Cushing syndrome with quality-of-life-reducing side effects like myopathy, sleeps disturbances, mood changes, or diabetes. Several case series suggested that the application of bevacizumab is safe in patients suffering from radionecrosis and improves the symptomatic burden in and thereby the quality of life [23, 24]. However, randomized prospective trials are needed to define the optimal scheduling, dosage, and duration of therapy.

In selected patients suffering from recurrent brain metastases not eligible for neurosurgical resection, stereotactic radiosurgery (including re-radiation), or whole-brain radiotherapy, but who are suffering from large peritumoral edema with mass effect (and the resulting quality-of-life-limiting symptoms), an experimental application of the anti-VEGF antibody bevacizumab could be considered. Here, case reports also report a restoration of functional independence and quality of life due to the control of symptoms and the reduction of steroid treatment [25]. Therefore, bevacizumab-based treatment might have, as shown for primary brain tumors (glioblastoma), a corticosteroid-sparing effect and thereby positively affect the patient’s health-related quality of life [26].

Further, anti-angiogenic therapies were postulated to have synergistic effects with whole-brain therapy. In line with this, the combination of bevacizumab and whole-brain radiotherapy was tested in a cohort mainly consisting of patients with metastatic breast cancer and brain metastases, was shown to be safe, and is currently being investigated in further trials [27]. Similarly, treatment with the VEGF receptor tyrosine kinase inhibitor sunitinib after stereotactic radiosurgery of 1–3 newly diagnosed brain metastases was investigated in a cohort of 14 patients with mixed histologies (lung, breast, melanoma) and showed a 6-month progression-free survival rate of 43%, further underling possible synergistic effects of anti-angiogenic therapies and radiotherapy [28].

## Conclusions

In conclusion, neo-angiogenesis is a specific hallmark of NSCLC brain metastasis development, while other histologies including breast and renal cell cancer might depend less on the induction of neo-angiogenesis. Therefore, neo-angiogenic therapies are a promising approach to prevent brain metastases in patients with advanced NSCLC. Several clinical studies suggest a therapeutic potential of bevacizumab-based treatments in patients with asymptomatic NSCLC brain metastases and inclusion of anti-angiogenic therapies should be evaluated in selected patients. A further important application of anti-angiogenic therapies is the treatment of symptomatic radiation necrosis or large, steroid-dependent peritumoral edema in patients without local therapy options. Further prospective clinical trials are needed to investigate the preventive as well as the therapeutic potential of anti-angiogenic therapies in patients with brain metastases.

**Table 1:** Selected clinical studies investigating bevacizumab-based treatment in brain metastasis patients

Entity	Combination Partner	Intracranial Response Rate (%)	PFS (months)	OS (months)	Ref
Asymptomatic nonsquamous NSCLC BM	Carboplatin, Paclitaxel (B + CP)	B + CP: 61.2	B + CP: 6.7	B + CP:16.0	[16]
	Erlotinib (B + E)	B + E: 12.5	B + E: 6.3	B + E: 12.0	
Asymptomatic nonsquamous NSCLC BM	Platinum based Chemotherapy	–	6.5	10.0	[17]
Asymptomatic nonsquamous NSCLC BM	Carboplatin, Pemetrexed	–	8.2	14.0	[15]
Colorectal carcinoma BM	After local treatment	–	–	20.6	[21]
	In combination with chemotherapy				

*NSCLC* non-small cell lung cancer, *BM* brain metastases, *B* *+* *CP* carboplatin + paclitaxel + bevacizumab, *B* *+* *E* erlotinib + bevacizumab, *PFS* progression-free survival, *OS* overall survival, *Ref* reference
